# Multimodal prediction of persistent pulmonary nodules after COVID-19: radiomics feature integration with clinical and epidemiologic variables

**DOI:** 10.3389/fmed.2026.1777725

**Published:** 2026-03-11

**Authors:** Lijuan Ma, Hongyuan Xiao, Yonggang Huang, Ru Nan, Yulong Ma, Xinru Liang

**Affiliations:** The First Affiliated Hospital of Hebei North University, Zhangjiakou, Hebei, China

**Keywords:** COVID-19, LASSO regression, nomogram, persistent nodules, predictive model, pulmonary nodules

## Abstract

**Background:**

Persistent pulmonary nodules are increasingly identified in patients recovering from coronavirus disease 2019 (COVID-19). However, factors associated with long-term persistence remain insufficiently understood.

**Objective:**

To determine whether a predictive model integrating clinical and CT imaging features can estimate the risk of pulmonary nodule persistence at 6 months after COVID-19.

**Methods:**

In this single-center retrospective cohort study, 419 patients with newly detected pulmonary nodules after confirmed COVID-19 infection who had ≥ 6 months of follow-up were included (January 2020–December 2024). Clinical and computed tomography (CT) features were collected. Predictors were selected using least absolute shrinkage and selection operator (LASSO) regression and incorporated into a multivariable logistic regression model. Model performance was assessed using receiver operating characteristic curves and calibration analysis. Internal validation was performed using 1,000 bootstrap resamples to estimate optimism-corrected performance. Decision curve analysis was also conducted.

**Results:**

Among 419 patients, 210 (50.1%) had persistent nodules at 6 months. In age- and sex-adjusted analyses, ≥ 4 hospitalizations, prior tuberculosis, larger maximum nodule diameter (OR per mm increase: 1.121, 95% CI: 1.074–1.170), vascular convergence sign positivity, and ICU admission were associated with persistence. LASSO selected four key predictors, and multivariable analysis confirmed ≥ 4 hospitalizations, prior tuberculosis, larger nodule diameter, and vascular convergence sign as independent risk factors. The model achieved an AUC of 0.728, with bootstrap-corrected AUC of 0.717. Decision curve analysis demonstrated clinical net benefit within threshold probabilities of 50–83%.

**Conclusion:**

The proposed clinical–imaging model effectively identifies patients at higher risk of persistent pulmonary nodules after COVID-19 and may assist in optimizing individualized follow-up strategies.

## 1 Introduction

Since outbreak in late 2019, coronavirus disease 2019 (COVID-19) has exerted sustained and profound impacts on global public health systems. Although most patients gradually recover after the acute infection stage, increasing evidence suggests that the pulmonary effects of COVID-19 may persist for months or even longer (1). Residual imaging abnormalities—such as ground-glass opacities, fibrotic streaks, and newly developed small nodules—may be present during recovery, indicating delayed or incomplete pulmonary repair processes (2, 3). Previous follow-up studies have reported that 20–40% of patients exhibit residual pulmonary lesions 6–12 months after infection, among which persistent nodules may arise from chronic inflammation, micro-fibrosis, or structural remodeling (3, 4).

Persistent pulmonary nodules following COVID-19 represent a post-infectious radiologic manifestation with uncertain clinical implications. Considerable heterogeneity exists among patients in terms of acute inflammatory severity, immune response, baseline lung structure, and comorbidities, leading to highly variable nodule absorption trajectories (5). Patients with a history of smoking, chronic obstructive pulmonary disease (COPD), or structural lung disease are more prone to incomplete resolution, while those with heightened inflammatory responses may experience delayed recovery (6, 7). In clinical practice, initial imaging findings and limited clinical information often fail to accurately predict nodule evolution, resulting in over-surveillance for some patients and insufficient follow-up for others.

Early identification of pulmonary nodules that are likely to persist is essential for optimizing post-COVID-19 follow-up strategies and improving allocation of medical resources. Persistent nodules may reflect ongoing inflammation or early fibrosis, and in patients with structural complexity, malignancy risks must also be considered (8). Therefore, integrating clinical variables with CT-derived imaging features may enable early risk stratification and support evidence-based clinical decision-making. Chest CT has been shown to reliably detect and characterize ground-glass opacities (GGO), which represent one of the most common imaging manifestations in COVID-19 (9). Recent radiomics and deep-learning studies further demonstrated that GGO patterns on CT can be quantitatively analyzed and incorporated into predictive modeling frameworks (10, 11). With advances in radiomics, quantitative imaging, and combined modeling approaches, predictive research has rapidly expanded in lung nodule characterization and COVID-19 imaging studies (12, 13). However, existing COVID-19-related diagnostic or prognostic models often display uncertain or high risk of bias—stemming from unrepresentative controls, exclusion of participants prior to outcome assessment, overfitting in model development, and incomplete reporting. Most studies also lack clearly defined target populations or intended scenarios and seldom examine calibration performance (13). Importantly, no systematic predictive studies specifically addressing the persistence of newly developed pulmonary nodules after COVID-19 are currently available.

Modern imaging techniques have played a pivotal role in the diagnosis and monitoring of COVID-19 (14). Chest computed tomography (CT) and conventional X-ray imaging have been widely used to demonstrate typical radiologic manifestations, including ground-glass opacities and vascular changes (15). Beyond morphologic assessment, emerging studies have emphasized the prognostic relevance of coagulation markers such as D-dimer and thromboplastin time in relation to imaging findings (16). Advances in imaging science—including ultrasound contrast media, nanoparticle-based CT contrast agents (17–19), phantom materials for CT calibration (20), and quality control of radiographic systems—have further improved imaging accuracy and reproducibility (21). In addition, ongoing evaluations of diagnostic imaging infrastructure and equipment acceptance testing highlight the importance of standardized acquisition protocols for reliable longitudinal assessment (22). Collectively, these developments provide a broader technical foundation for imaging-based predictive modeling in post-COVID pulmonary evaluation.

Therefore, this study aimed to identify major factors influencing nodule persistence using LASSO regression for variable selection and multivariable logistic regression for model development. We sought to establish a robust and clinically applicable risk-prediction tool to support individualized follow-up strategies and streamline post-COVID-19 surveillance.

## 2 Materials and methods

### 2.1 Study design and population

This single-center retrospective cohort study investigated imaging evolution and predictors of persistent pulmonary nodules after COVID-19. Eligible participants were inpatients with COVID-19 at The First Affiliated Hospital of Hebei North University between January 2020 and December 2024 who underwent chest CT during recovery and were identified with newly developed pulmonary nodules.

### 2.2 Inclusion and exclusion criteria

Patients were included if they had newly detected nodules on baseline CT following COVID-19 infection, underwent at least one follow-up CT examination ≥ 6 months later, and had adequate imaging quality for evaluation. Patients were excluded if they had prior lung malignancy or active pulmonary tuberculosis, incomplete imaging data, missing key CT sequences, or baseline and follow-up scans unsuitable for comparison. Radiologic evidence of old healed tuberculosis was carefully distinguished from newly developed post-COVID nodules based on prior imaging records and lesion morphology to minimize misclassification bias.

### 2.3 Variable definitions

#### 2.3.1 Clinical variables

Demographic and clinical data were collected, including age, sex, smoking history, chronic obstructive pulmonary disease (COPD), prior tuberculosis, diabetes, acute COVID-19 severity, number of hospitalizations, intensive care unit (ICU) admission, vaccination status, co-infection during treatment, and peak C-reactive protein (CRP-peak) levels.

#### 2.3.2 Imaging variables

Two experienced thoracic radiologists independently reviewed CT scans, with discrepancies resolved by a third radiologist. Imaging features included: affected lobe, subpleural location, nodule type (pure ground-glass, part-solid, or solid), maximum nodule diameter, spiculation, lobulation, air bronchogram sign, and vascular convergence sign (VC).

#### 2.3.3 Primary outcome

The primary outcome, Persistent 6-month, was defined as visible nodules at ≥ 6-month follow-up with < 2 mm change in maximum diameter or < 25% change in volume, based on international nodule follow-up guidelines (23, 24).

### 2.4 Statistical analysis

Continuous variables were expressed as mean ± SD or median (IQR) according to distribution, and categorical variables as counts and percentages.

Model development followed a two-step strategy to reduce overfitting while preserving clinical interpretability. First, least absolute shrinkage and selection operator (LASSO) regression with 10-fold cross-validation was applied for variable selection, retaining predictors with non-zero coefficients at the optimal penalty parameter. Selected variables, together with clinically relevant covariates, were then entered into a multivariable logistic regression model to derive adjusted effect estimates and construct the final prediction model.

Model discrimination was quantified using the area under the receiver operating characteristic curve (AUC). Internal validation of discrimination was performed using 1,000 bootstrap resamples; in each iteration, the model was refitted and applied to the original dataset to estimate the distribution and variability of AUC values, thereby assessing model optimism and stability.

Calibration was evaluated using calibration plots comparing predicted and observed probabilities and statistically assessed with the Hosmer–Lemeshow goodness-of-fit test. Clinical utility was assessed using decision curve analysis (DCA) across a range of threshold probabilities. To minimize optimism in net benefit estimation, DCA was conducted using cross-validated predicted probabilities and further supported by bootstrap resampling.

Variables with < 5% missing data were imputed using median substitution. For variables with ≥ 5% missingness, multiple imputation by chained equations (five imputations) was performed under the assumption of missing at random. All analyses were performed using R version 4.4.3, and a two-sided *P* < 0.05 was considered statistically significant.

## 3 Results

### 3.1 Participant characteristics

Among the 419 included patients, 209 (49.9%) showed complete nodule resolution and 210 (50.1%) exhibited persistence at 6 months. Baseline characteristics are shown in Table 1. No significant differences were found in age, sex, number of hospitalizations, smoking history, COPD, co-infection, vaccination status, diabetes, lobe distribution, subpleural location, nodule type, spiculation, lobulation, or air bronchogram (all *P* > 0.05). Prior tuberculosis was more common in the persistence group (9.6% vs. 3.8%, *P* = 0.030). The positivity rates of VC and tuberculosis history were significantly higher in the persistence group (*P* < 0.001, *P* = 0.030). Maximum nodule diameter was also significantly larger in persistent nodules (12.83 ± 5.63 mm vs. 10.12 ± 4.06 mm, *P* < 0.001).

**TABLE 1:** Baseline characteristics of patients with persistent and non-persistent pulmonary nodules at 6-month follow-up.

Variable	Non-persistent group (*n* = 209)	Persistent group (*n* = 210)	*t/z/x* ^2^	*P*
Gender			0.062	0.803
Man	115 (55.0%)	112 (53.3%)		
Female	94 (45.0%)	98 (46.7%)		
Age	65.38 (11.16)	64.27 (11.91)	0.985	0.325
Number of hospitalizations			3.798	0.150
1 time	150 (71.8%)	142 (67.6%)		
2–3 times	50 (23.9%)	49 (23.3%)		
≥ 4 times	9 (4.3%)	19 (9.0%)		
Smoking			4.357	0.113
Never	113 (54.1%)	133 (63.3%)		
Former	71 (34.0%)	61 (29.0%)		
Current	25 (12.0%)	16 (7.6%)		
COPD history			2.910	0.088
No	166 (79.4%)	181 (86.2%)		
Yes	43 (20.6%)	29 (13.8%)		
Tuberculosis history			4.687	0.030
No	202 (96.2%)	189 (90.4%)		
Yes	8 (3.8%)	20 (9.6%)		
COVID-19 infection during treatment			0.020	0.887
No	188 (90.0%)	187 (89.0%)		
Yes	21 (10.0%)	23 (11.0%)		
Vaccinated			0.193	0.660
No	102 (48.8%)	108 (51.4%)		
Yes	107 (51.2%)	102 (48.6%)		
Diabetes			0.100	0.752
No	161 (77.0%)	158 (75.2%)		
Yes	48 (23.0%)	52 (24.8%)		
Lung lobe			0.370	0.831
Upper	65 (31.1%)	70 (33.3%)		
Middle	72 (34.4%)	73 (34.8%)		
Lower	72 (34.4%)	67 (31.9%)		
Subpleural location			0.585	0.444
No	76 (36.4%)	85 (40.5%)		
Yes	133 (63.6%)	125 (59.5%)		
Nodule type			0.476	0.788
Pure GGO	49 (23.4%)	45 (21.4%)		
Part-solid	38 (18.2%)	43 (20.5%)		
Solid	122 (58.4%)	122 (58.1%)		
Spiculation			0.061	0.805
No	148 (70.8%)	152 (72.4%)		
Yes	61 (29.2%)	58 (27.6%)		
Lobulation			3.219	0.073
No	12 (5.7%)	4 (1.9%)		
Yes	197 (94.3%)	206 (98.1%)		
Air bronchogram sign			2.888	0.089
No	10 (4.8%)	3 (1.4%)		
Yes	199 (95.2%)	207 (98.6%)		
Vascular convergence sign (VC)			25.866	< 0.001
No	145 (69.4%)	93 (44.3%)		
Yes	64 (30.6%)	117 (55.7%)		
ICU admission			3.535	0.060
No	188 (90.0%)	200 (95.2%)		
Yes	21 (10.0%)	10 (4.8%)		
Peak CRP (mg/L)	40.97 (31.78)	36.99 (34.60)	1.225	0.221
Maximum nodule diameter (mm)	10.12 (4.06)	12.83 (5.63)	–5.649	< 0.001
Length of hospital stay (days)	10.55 (8.11)	10.23 (7.09)	0.426	0.671

### 3.2 Age- and sex-adjusted logistic regression analysis for predictors of persistent pulmonary nodules

The results of the age- and sex-adjusted logistic regression analyses are presented in Table 2. After adjusting for age and sex, patients with ≥ 4 hospitalizations had an increased risk of persistent pulmonary nodules (*OR* = 2.322, *95% CI*: 1.013–5.324, *P* = 0.047). Maximum nodule diameter, analyzed as a continuous variable, was significantly positively associated with nodule persistence *(OR* = 1.121, *95% CI*: 1.074–1.170, *P* < 0.001). The presence of the vascular convergence (VC) sign markedly increased the risk of nodule persistence (*OR* = 2.825, 95% CI: 1.891–4.221, *P* < 0.001) and a history of pulmonary tuberculosis was significantly associated with an increased risk of persistence (*OR* = 2.671, 95% CI: 1.151–6.201, *P* = 0.031). In addition, a history of ICU admission showed a protective effect (*OR* = 0.453, 95% CI: 0.208–0.990, *P* = 0.047). Other variables—including sex, smoking history, COPD, vaccination status, diabetes, subpleural location, nodule type, spiculation, and lobulation—were not significantly associated with persistent nodules.

**TABLE 2:** Age- and sex-adjusted logistic regression analysis for predictors of persistent pulmonary nodules.

Variable	OR (95%CI)	*P*
**Number of hospitalizations**		
1 time	1	
2–3 times	1.054 (0.665–1.669)	0.823
≥ 4 times	2.322 (1.013–5.324)	0.047
**Smoking**		
Never	1	
Former	0.687 (0.426–1.106)	0.122
Current	0.494 (0.240–1.019)	0.056
**COPD history**		
No	1	
Yes	0.639 (0.378–1.079)	0.094
**Tuberculosis history**		
No	1	
Yes	2.671(1.151–6.201)	0.031
**COVID-19 infection during treatment**		
No	1	
Yes	1.123 (0.599–2.105)	0.717
**Vaccinated**		
No	1	
Yes	0.907 (0.618–1.331)	0.617
**Diabetes**		
No	1	
Yes	1.134 (0.721–1.783)	0.587
Peak CRP (mg/L)	0.996 (0.990–1.002)	0.196
**Lung lobe**		
Upper	1	
Middle	0.939 (0.587–1.502)	0.794
Lower	0.858 (0.533–1.381)	0.529
**Subpleural location**		
No	1	
Yes	0.862 (0.579–1.283)	0.464
**Nodule type**		
Pure GGO	1	
Part-solid	1.302 (0.712–2.380)	0.392
Solid	1.122 (0.694–1.812)	0.638
Maximum nodule diameter (mm)	1.121 (1.074–1.170)	< 0.001
**Spiculation**		
No	1	
Yes	0.914 (0.597–1.400)	0.679
**Lobulation**		
No	1	
Yes	3.104 (0.983–9.799)	0.053
**Air bronchogram sign**		
No	1	
**Vascular convergence sign (VC)**		
Yes	3.371 (0.912–12.453)	0.068
No	1	
Yes	2.825 (1.891–4.221)	< 0.001
**ICU admission**		
No	1	
Yes	0.453 (0.208–0.990)	0.047

### 3.3 Variable selection using LASSO regression

**FIGURE 1:**
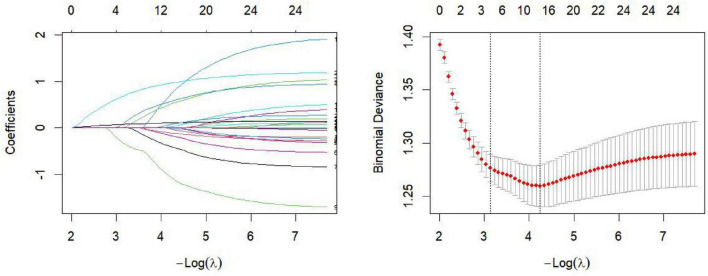
LASSO coefficient profiles and cross-validation curve for variable selection. Binomial deviance represents model fit under the binomial distribution, and coefficients refer to the estimated parameter weights. The cross-validation error plot (right) displays the cross-validated deviance across different log-lambda values, assisting in selecting the optimal regularization parameter to improve model generalizability. Age and sex were adjusted for in the LASSO model and were not penalized. The coefficient path plot (left) illustrates how the coefficients of individual predictors change as the regularization strength (log-lambda) increases. Predictors with non-zero coefficients are considered important, whereas those with coefficients shrunk to zero contribute minimally to the model. Each colored line in this figure represents a distinct predictor.

All candidate variables were entered into the least absolute shrinkage and selection operator (LASSO) regression model for predictor selection. The results identified *number of hospitalizations* (β = 0.083), *tuberculosis history* (β = 0.319), *maximum nodule diameter* (β = 0.072), and *vascular convergence (VC) sign* (β = 0.677) as variables associated with the outcome. Coefficients of all other variables shrank to zero due to strong penalization, indicating minimal contribution to outcome prediction (Figure 1). Although smoking history and COPD are clinically relevant factors, they were not retained in the final model because their coefficients shrank to zero in the LASSO procedure, suggesting limited incremental predictive value after accounting for other variables.

### 3.4 Multivariable logistic regression analysis for predictors of persistent pulmonary nodules

Variables selected through LASSO regression were further analyzed in a multivariable logistic regression model (Table 3). After adjusting for age and sex, maximum nodule diameter remained an independent risk factor for nodule persistence (*OR* = 1.128, 95% CI: 1.078–1.180, *P* < 0.001). Patients with ≥ 4 hospitalizations continued to exhibit a significantly higher risk of persistent nodules (*OR* = 2.660, 95% CI: 1.084–6.530, *P* = 0.033). VC positivity significantly increased the likelihood of persistence (*OR* = 3.200, 95% CI: 2.080–4.923, *P* < 0.001). Prior tuberculosis remained an independent risk factor for nodule persistence in the multivariable model (*OR* = 2.302, 95% CI: 1.122–4.801, *P* < 0.001). Neither age nor sex showed statistical significance (both *P* > 0.05).

**TABLE 3:** Multivariable logistic regression analysis for predictors of persistent pulmonary nodules.

Variable	OR (95%CI)	*P*
Age	0.993 (0.975–1.011)	0.427
**Gender**		
Man	1	
Female	0.987 (0.648–1.505)	0.953
**Number of hospitalizations**		
1 time	1	
2–3 times	1.158 (0.701–1.913)	0.565
≥ 4 times	2.660 (1.084–6.530)	0.033
**Tuberculosis history**		
No	1	
Yes	2.302 (1.122–4.801)	< 0.001
Maximum nodule diameter (mm)	1.128 (1.078–1.180)	< 0.001
**Vascular convergence sign (VC)**		
No	1	
Yes	3.200 (2.080–4.923)	< 0.001

Multivariable logistic regression was performed. All variables with non-zero coefficients identified by LASSO regression were entered into the model.

### 3.5 Evaluation of the predictive model

**FIGURE 2:**
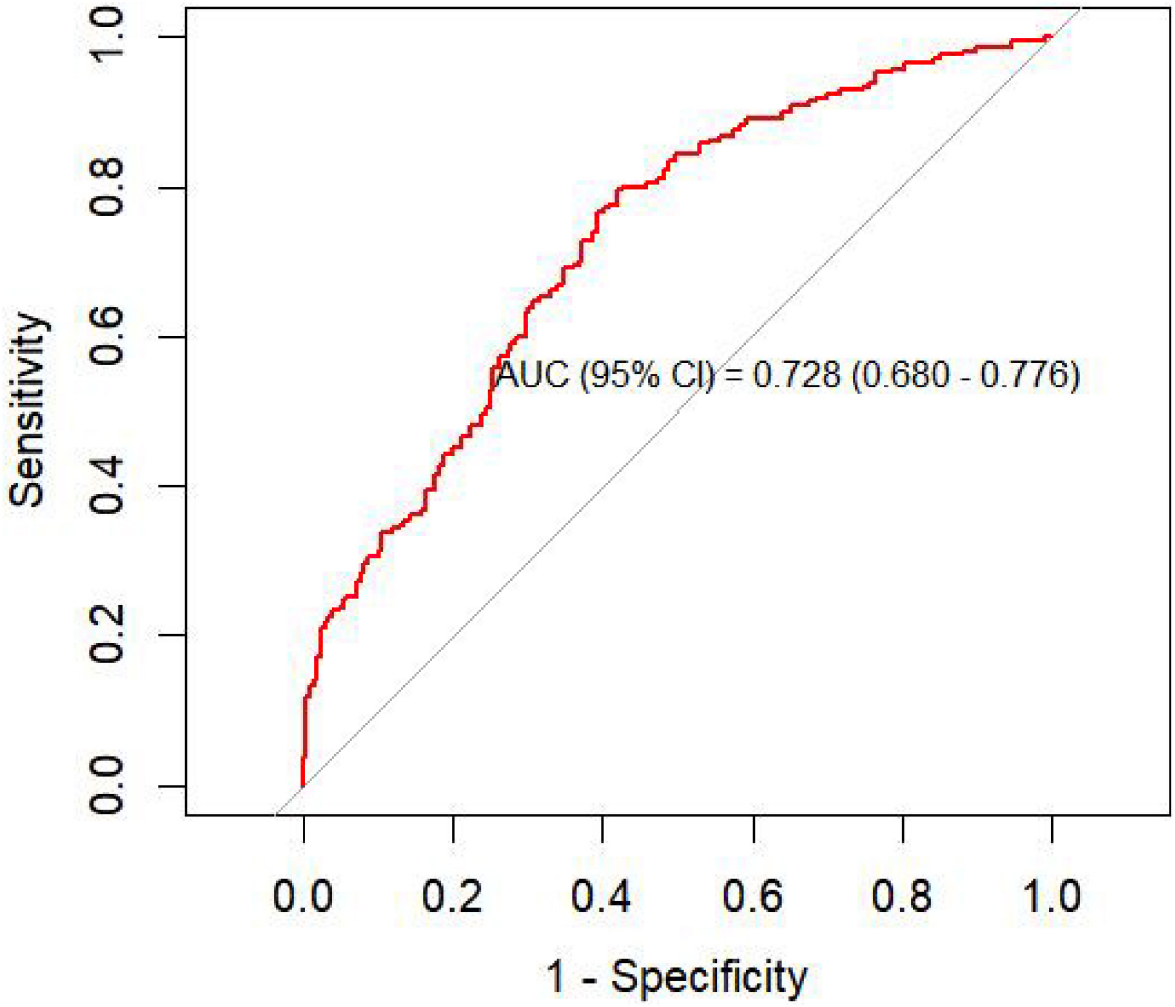
ROC curve of the prediction model in the training cohort.

To assess the discriminative performance of the predictive model, a receiver operating characteristic (ROC) curve was first generated. The model demonstrated good ability to distinguish nodules that persisted at ≥ 6 months from those that resolved, with an AUC = 0.728 (95% CI: 0.680–0.776) (Figure 2).

**FIGURE 3:**
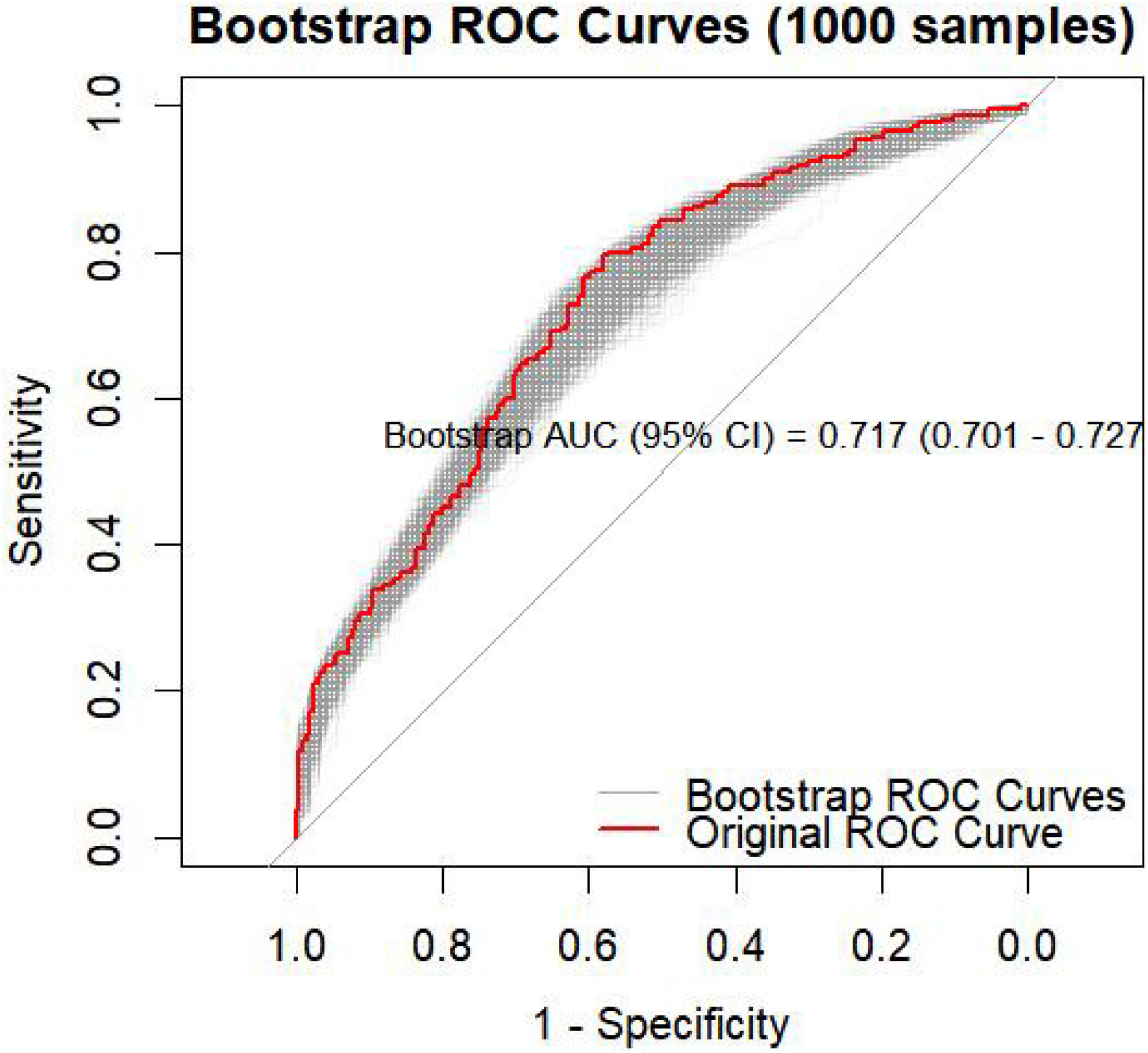
Bootstrap ROC curves (1,000 resampling iterations) with bootstrap AUC (95% CI).

To further evaluate the robustness of the model, internal validation was performed using 1,000 bootstrap resampling iterations. The bootstrap-corrected mean AUC = 0.717 (95% CI: 0.701–0.727) (Figure 3), closely approximating the original model’s AUC. The overall shape of the bootstrapped ROC curves remained stable without marked fluctuations, indicating excellent internal consistency and suggesting a strong generalization capacity of the model.

### 3.6 Construction and validation of the nomogram and clinical decision curve

A nomogram was constructed based on the independent predictors identified in the multivariable logistic regression analysis to estimate the probability of 6-month nodule persistence. By integrating clinical characteristics with imaging features, the model enabled individualized and quantitative risk assessment. The discriminative ability of the model was first evaluated using receiver operating characteristic (ROC) analysis. The ROC curve demonstrated good discrimination, with an AUC (95% CI) of 0.728 (0.680–0.776) (Figure 2). Internal validation using 1,000 bootstrap resampling iterations showed a stable AUC of 0.717 (95% CI: 0.701-0.727), indicating robust and reliable model performance (Figure 3).

**FIGURE 4:**
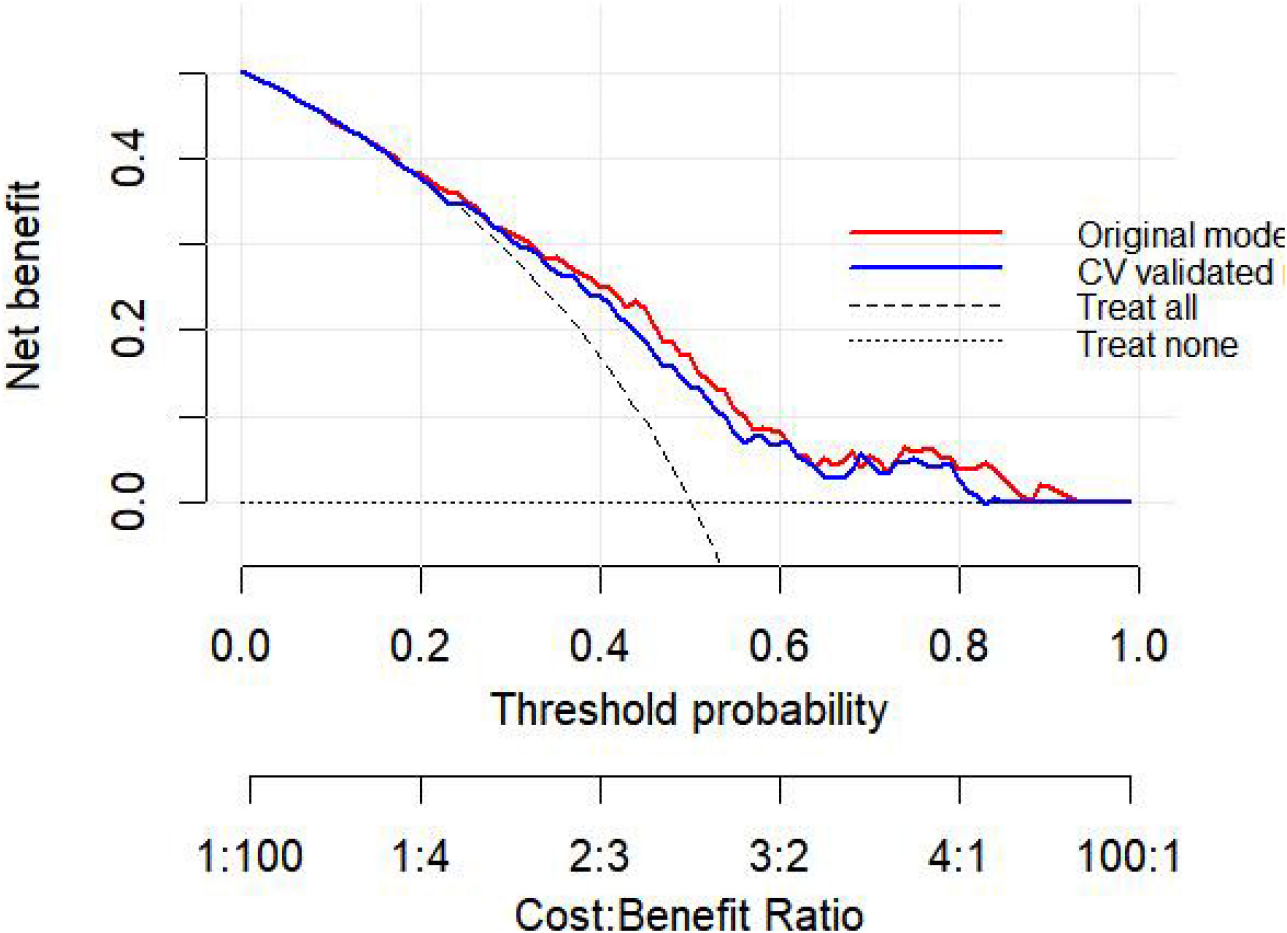
Decision curve analysis (DCA) of the prediction model for persistent pulmonary nodules at 6 months.

**FIGURE 5:**
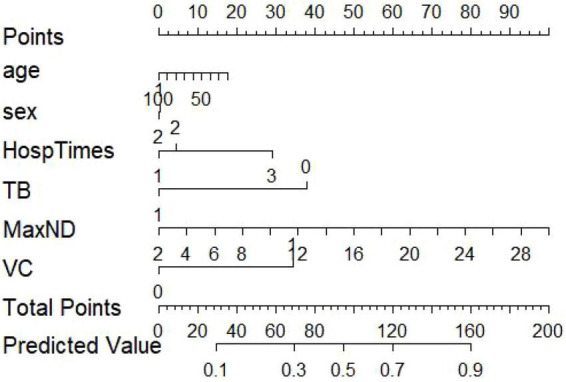
Nomogram for predicting the probability of persistent pulmonary nodules at 6 months. TB, Tuberculosis history; MaxND, Maximum nodule diameter (mm); VC, Vascular convergence sign.

**FIGURE 6:**
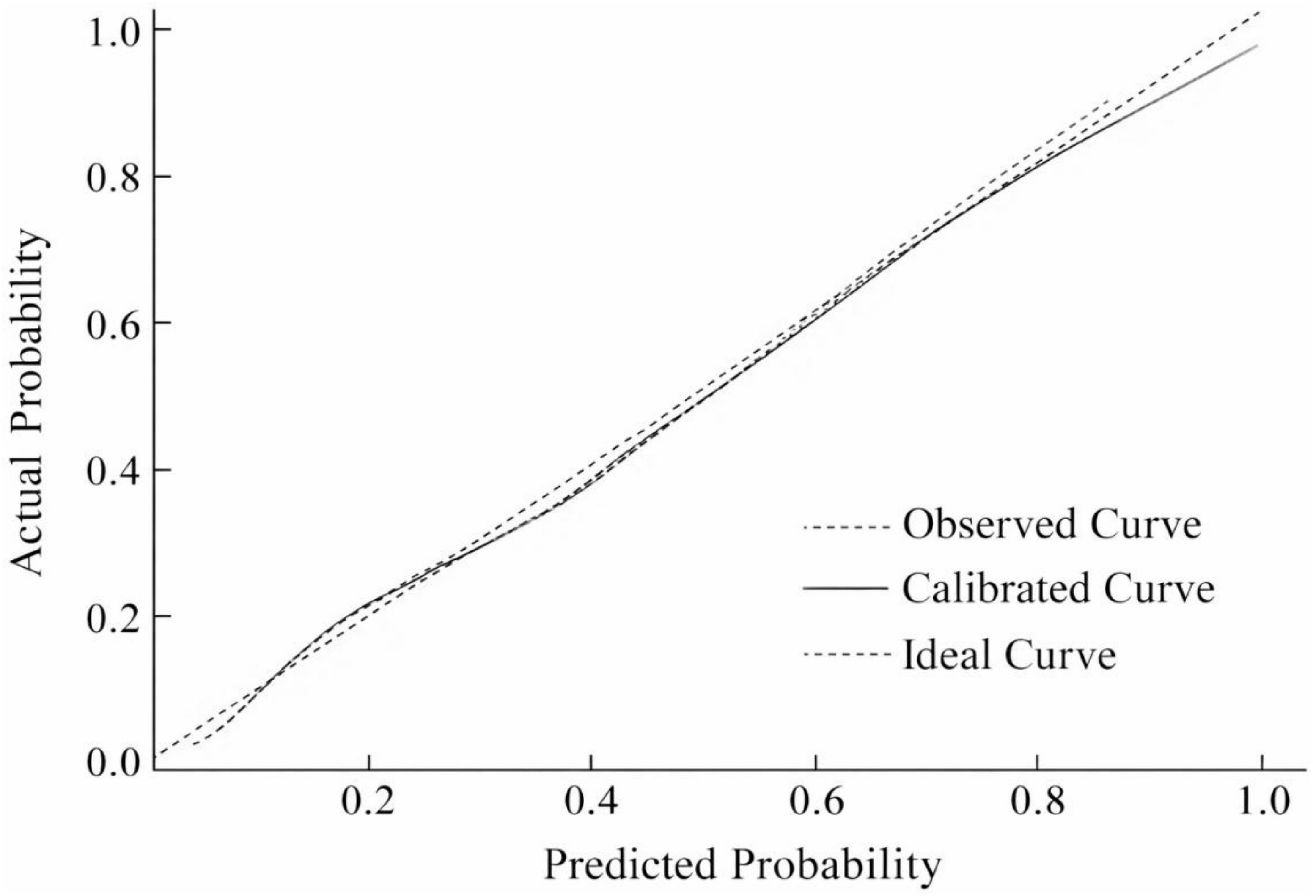
Calibration curve of the prediction model.

Clinical decision curve analysis (DCA) was subsequently performed to evaluate the clinical utility of the prediction model. The results indicated that within a threshold probability range of approximately 50-83%, the model provided greater net benefit than both the “treat-all” and “treat-none” strategies, suggesting potential clinical usefulness in identifying patients at high risk of persistent pulmonary nodules (Figure 4). Based on the identified predictors, a nomogram was then developed to facilitate individualized prediction of persistent pulmonary nodules at 6 months (Figure 5). Calibration curve analysis demonstrated good agreement between predicted and observed probabilities. The calibration plot closely followed the ideal 45° reference line, and the Hosmer-Lemeshow test showed no evidence of poor fit (*P* > 0.05), supporting the reliability of the model (Figure 6).

## 4 Discussion

This study analyzed 419 patients who developed new-onset pulmonary nodules following COVID-19 infection and completed at least 6 months of radiologic follow-up. Using LASSO regression combined with multivariable logistic regression, we developed and internally validated a prediction model integrating both clinical and imaging characteristics. Four variables—number of hospitalizations, prior tuberculosis, maximum nodule diameter, and the presence of the vascular convergence (VC) sign—were ultimately included in the final model. The model demonstrated favorable discrimination and calibration. After adjustment for age and sex, ≥ 4 hospitalizations, larger nodule diameter, prior tuberculosis, and VC positivity remained significantly associated with an increased risk of nodule persistence.

In recent years, research on residual pulmonary imaging abnormalities after COVID-19 has grown rapidly. Follow-up studies have shown that approximately 20–40% of patients continue to exhibit varying degrees of residual imaging abnormalities 6–12 months after infection, including ground-glass opacities, linear opacities, and new or persistent small nodules (25, 26). The formation of persistent nodules may be closely related to chronic inflammation, micro-fibrosis, and incomplete repair processes (27), which aligns with our finding that “larger nodules are more likely to persist.” Furthermore, our study identified VC positivity as one of the strongest predictors. This is highly consistent with previous reports describing vascular traction, remodeling, and local inflammatory exudation–induced vascular clustering in residual post-COVID lesions (9, 28). Some studies have suggested that the vascular convergence sign may reflect a focal traction effect caused by inflammatory lesions and alveolar structural disruption, indicating that the local lesions may be more prone to prolonged non-resolution (29).

The number of hospitalizations was another important determinant in our study. Patients with ≥ 4 hospitalizations were significantly more likely to demonstrate persistent nodules, underscoring the potential impact of higher disease burden during the acute phase of COVID-19. Frequent hospitalizations often reflect more severe inflammation, higher viral load, or the presence of multiple comorbidities, all of which may contribute to more extensive parenchymal injury and slower healing. This is consistent with prior studies showing that severe COVID-19 patients exhibit more persistent CT abnormalities during longitudinal follow-up (30, 31). Mechanistically, COVID-19 can induce diffuse alveolar damage, microvascular injury, and activation of profibrotic pathways (32). Thus, persistent nodules may represent residual inflammation, organizing pneumonia, granulation tissue proliferation, or early fibrosis—patterns similarly documented in long-term studies of SARS and MERS (33). Additionally, VC typically suggests ongoing inflammatory exudation, fibrotic traction, or architectural remodeling, constituting a common but often persistent post-infectious imaging manifestation (34). A history of pulmonary tuberculosis may further reshape the pulmonary immune microenvironment, altering macrophage and T-cell responses and potentially modulating the inflammatory intensity and repair efficiency during subsequent viral infections (35). Biochemical and hematological factors may also contribute to prolonged nodule persistence after COVID-19. In our study, peak CRP levels did not significantly differ between groups, suggesting that systemic inflammatory burden during the acute phase may not be the sole determinant of long-term radiologic evolution. Previous longitudinal studies have shown that persistent radiologic abnormalities at 6 months are more closely related to sustained inflammatory signaling—particularly interleukin-6 (IL-6)—and to systemic disease severity rather than CRP alone (36). In addition, COVID-19–associated endothelial injury and coagulation abnormalities may promote microvascular dysfunction and localized fibrotic repair, thereby delaying lesion resolution (37, 38). These mechanisms are biologically consistent with imaging features such as the vascular convergence sign observed in our model. Future studies incorporating broader laboratory markers, including IL-6 and coagulation indices, may further refine risk stratification. Collectively, conditions that may predispose to long-term nodule persistence include severe or recurrent inflammatory insults during acute infection, repeated hospitalizations reflecting higher disease burden, pre-existing structural lung abnormalities, and altered pulmonary immune microenvironments such as those seen in prior tuberculosis (39, 40). These factors may converge to promote sustained inflammatory signaling, dysregulated tissue repair, microvascular remodeling, and localized profibrotic responses (41), ultimately contributing to delayed radiologic resolution and persistent nodular manifestations.

The prediction model demonstrated robust internal validation performance, with ROC analysis, calibration curves, and decision curve analysis confirming its reliability and clinical utility. In clinical follow-up, accurate identification of high-risk nodules may help reduce unnecessary repeated imaging while ensuring that patients at elevated risk receive appropriate monitoring. The nomogram provides a practical and accessible tool for individualized risk assessment, particularly valuable in primary care settings or regions with limited imaging resources.

Despite these strengths—including a relatively large sample size, a rigorously validated modeling framework with cross-validation and bootstrap resampling, and comprehensive evaluation of discrimination, calibration, and clinical utility—several limitations should be acknowledged. First, the single-center retrospective design may introduce selection bias and limit external generalizability. Second, we defined nodule persistence based on a 6-month follow-up interval; however, some post-infectious nodules may require longer observation to fully resolve, suggesting the need for extended follow-up. Third, we included only conventional imaging features and did not incorporate radiomics or deep-learning features, which may restrict further model refinement. Moreover, the absence of pathological confirmation prevented elucidation of the precise histopathological basis of persistent nodules. Future studies should include multicenter external validation and integrate radiomics, AI-assisted analysis, and longitudinal imaging data to develop more precise and clinically generalizable predictive tools. Although patients with active tuberculosis were excluded, individuals with a prior history of tuberculosis were retained in the analysis. Structural lung alterations related to healed tuberculosis may partially overlap with post-COVID imaging findings, potentially influencing persistence assessment despite careful radiologic differentiation.

## 5 Conclusion

In conclusion, we constructed and validated a prediction model integrating clinical and baseline imaging characteristics to estimate the risk of persistent new-onset pulmonary nodules 6 months after COVID-19 infection. Number of hospitalizations, maximum nodule diameter, vascular convergence sign, and prior pulmonary tuberculosis were key determinants of persistence. The nomogram demonstrated great predictive performance, stability, and potential clinical applicability, providing a quantitative basis for developing follow-up strategies in patients recovering from COVID-19.

## Data Availability

The raw data supporting the conclusions of this article will be made available by the authors, without undue reservation.
